# Management and burden of disease in people with myelin oligodendrocyte glycoprotein antibody-associated disease: data from an international, cross-sectional survey

**DOI:** 10.1007/s00415-025-13233-7

**Published:** 2025-07-22

**Authors:** F. Paul, S. Zappacosta, S. Narduzzi, M. Khellaf, M. Unsworth, E. Trenholm, M. Levy

**Affiliations:** 1 Experimental and Clinical Research Center, Department of Clinical Neuroimmunology, Charité,—Universitätsmedizin Berlin and Max Delbrueck Center for Molecular Medicine, Berlin, Germany; 2 UCB, Bulle, Switzerland; 3 UCB, Breda, Netherlands; 4 UCB, Brussels, Belgium; 5 Adelphi Real World, Bollington, UK; 6 Department of Neurology, Massachusetts General Hospital,, Harvard Medical School, Boston, MA USA

**Keywords:** Myelin-oligodendrocyte glycoprotein (MOG) antibody-associated disease (MOGAD), Cross-sectional studies, Disease management, Treatment patterns, Disease burden, Quality of life

## Abstract

**Background:**

There are challenges in the diagnosis of myelin-oligodendrocyte glycoprotein (MOG) antibody-associated disease (MOGAD), and a current lack of targeted treatments. This study investigated the disease management and burden of MOGAD in a real-world setting.

**Methods:**

Data were derived from the Adelphi MOGAD Disease Specific Programme (DSP)™, a cross-sectional survey of neurologists and their consulting patients with MOGAD, conducted in Europe and the United States in 2022. Neurologists reported on patient demographics, clinical characteristics, disease management history, treatments prescribed and burden of disease. Patients voluntarily reported on their perceptions on burden of disease. All analyses were descriptive.

**Results:**

Overall, 74 neurologists provided data for 268 consecutively consulting patients with MOGAD, of whom 66 completed voluntary questionnaires. Sixty four percent of patients received a preliminary/alternative diagnoses, and patients underwent a median (Q1, Q3) of 12.0 (9.0; 19.0) blood tests, assessments and/or scans to confirm MOGAD diagnosis. The median (interquartile range, Q1, Q3) physician-reported time from symptom onset to preliminary/alternative diagnosis was 19.0 (0.0; 59.0) days, and from symptom onset to definitive diagnosis 64.0 (31.0; 150.2) days. At time of the survey, 91.8% and 83.5% of patients were prescribed acute and maintenance treatment, respectively. Symptomatic burden remained moderately high, with patients reporting quality of life (QoL) and work productivity impairments.

**Conclusion:**

Patients with MOGAD may suffer from challenges in diagnosis, and disease management remains suboptimal, with burden to patients affecting their QoL and ability to work. Both the diagnosis and treatment of MOGAD should continue to be the subject of further research.

**Supplementary Information:**

The online version contains supplementary material available at 10.1007/s00415-025-13233-7.

## Introduction

Myelin-oligodendrocyte glycoprotein (MOG) antibody-associated disease (MOGAD) is a neuroinflammatory, demyelinating disorder of the central nervous system associated with autoantibodies to MOG in the serum [1–3]. MOGAD is considered a rare disease, with a prevalence of approximately 1.3–2.5/100,000, and an estimated annual incidence of approximately 3.4–4.8/1,000,000 [4].

MOGAD most commonly presents in adults as optic neuritis and transverse myelitis, however other clinical phenotypes include acute disseminated encephalomyelitis (ADEM), brainstem or cerebellar features, or cerebral cortical encephalitis [5–8]. The course of MOGAD may be monophasic or relapsing; reported proportions of patients with relapsing MOGAD vary widely among studies with a range of approximately 20–90%, and in individual patients the prediction of further attacks and accrual of disability is difficult [5–7, 9–14]. The clinical burden of MOGAD attacks can be severe, including optical and myelitic symptoms. Meningeal symptoms can also occur, including encephalopathy, seizures and focal deficits, and cerebral cortical encephalitis may also occur [15]. Patients also frequently experience pain, depression, cognitive defects, and worsened quality of life throughout the course of the disease [16–19].

Given the diversity in symptomatic presentation, the diagnosis of MOGAD presents challenges, although the diagnostic landscape is currently evolving. MOGAD has clinical and radiologic characteristics in common with multiple sclerosis (MS) and aquaporin-4 antibody-associated neuromyelitis optical spectrum disorder (NMOSD) [3, 20]. The discovery of aquaporin-4 and, subsequently, MOG antibodies has only recently characterized these three diseases as separate conditions [21–23]. Widespread awareness of MOGAD as a distinct disease is still lacking [2]; however international consensus diagnostic criteria for MOGAD (the 2023 Banwell criteria) have recently been published [15], followed shortly after by a diagnosis code for MOGAD in the International Classification of Diseases, 10th Revision, Clinical Modification (ICD-10-CM) [24].

There are currently no approved treatments for MOGAD. Treatments for acute attacks/relapses typically involve intravenous (IV) corticosteroids followed by oral corticosteroids, with IV human immunoglobulins (IVIg), and plasma exchange (PLEX) also being used [25, 26]. Preventative/maintenance treatment to prevent relapses also frequently includes corticosteroids, rituximab, azathioprine, mycophenolate, tocilizumab and IVIg [25, 27–31]. The use of corticosteroids has been associated with a broad side-effect burden, including infections, gastric disorders, fractures and psychiatric episodes, leading to increased financial burden [32, 33].

Quality real-world data can aid in characterizing the clinical profile and burden of patients diagnosed with rare diseases, as well as further understanding their treatment pathways. In light of the challenges on the MOGAD diagnosis and lack of targeted treatments to date, the aim of the current study was to generate insights on the disease management and burden of MOGAD. We present real-world survey data from neurologists and their patients with MOGAD to address this objective.

## Methods

### Study design

This study was an analysis of secondary data from the Adelphi MOGAD Disease Specific Programme (DSP)™. DSPs are large, multinational, independent cross-sectional surveys which collect information on real-world clinical practice and are designed to provide robust data on disease management, physician and patient attitudes, and the clinical profile and disease burden of patients. The methodology has been previously described, [34, 35], validated [36], and demonstrated to be representative and consistent over time [37].

This study includes data from neurologists and their consulting patients with MOGAD, with data collected in Europe (EU5; France, Germany, Italy, Spain and the United Kingdom) and the United States (US) between June and November 2022.

Neurologists were eligible to participate in the DSP provided they were directly involved in the treatment and management of ≥ 1 patient with MOGAD. They were each invited to complete an online survey, capturing their perceptions and attitudes toward the management of MOGAD. Neurologists were recruited to participate in the DSP by local fieldwork agents, and the data collection setting was secondary neurology services (public or private hospitals, clinics, or offices).

Neurologists completed online physician-completed patient record forms (PRFs) for up to their next 10 consecutively consulting patients with a physician-confirmed MOGAD diagnosis, to generate a patient sample representative of patients with MOGAD presenting in real-world clinical practice at the time the survey was conducted. Patients were at different points in their diagnostic and treatment journey. Cross sectional data were collected at the point of patient consultation, with retrospective data also collected from patient medical records. No follow-up data were collected.

Physicians provided comprehensive information on demographics; the patient journey from first symptoms to disease monitoring (i.e., timing and characteristics at symptom onset, alternative and/or definitive diagnosis and start of disease management); comorbidities, symptom severity (3-point scale); and burden of disease, including presenting symptoms, number of relapses, and hospitalisations. Neurologists were asked to report how much they believed that the patient’s MOGAD limited their physical functioning (5-point scale), social functioning (5-point scale), emotional wellbeing (5-point scale), as well as rating the patient’s overall quality of life (7-point scale).

With respect to treatments, neurologists reported the acute treatment administered during the patient’s first symptomatic episode included the type of treatment, the date that treatment started and ended, the dose given and if there were any amends. Details on long-term maintenance treatment administered were also collected, included the type of treatment, the date that treatment started and ended, the dose given, frequency of treatment and if there were any amends.

Completion of the PRF was undertaken through consultation of existing patient clinical records, as well as the judgment and diagnostic skills of the respondent physician, which reflects decisions made in routine clinical practice.

Patients were eligible for inclusion if they were aged ≥ 18 years at the time of the survey, had a physician-confirmed diagnosis of MOGAD, and visited a participating neurologist. Following informed consent, each patient for whom a neurologist completed a PRF was invited to complete a voluntary patient self-completion form (PSC), reporting on their perceptions around the burden of disease. The PSC included, but was not restricted to, a number of formally validated instruments including the Work Productivity and Activity Impairment (WPAI) [38] and the Medical Outcomes Study 36-item Short-Form Health Survey (SF-36) [39–41], assessing the effect of health problems on work productivity and regular activities; and various domains of physical and mental health, respectively. PSCs were completed independently from the physician and were returned in a sealed envelope, ensuring the patient’s responses were kept confidential from physicians.

### Data analysis

As the primary objective of this study was descriptive, and this study was an analysis of existing secondary data, no formal sample size calculations were performed. The sample size was determined by the duration of the original survey period and the willingness of the physicians and their patients to participate. Data are presented descriptively, and no statistical comparisons were performed.

Continuous data are presented as mean, standard deviation (SD), median and interquartile range (Q1, Q3), as well as minimum and maximum values. For categorical data, counts and proportions of observations for each of the categories are presented, with missing data (don’t know response or non-response) reported as a separate category where applicable. Completion of all questions was not always possible, as physicians could only report data available to them at the time of patient consultation or retrospectively available in the patient medical records, and completion of the patient-reported PSC was voluntary. Therefore, the base of patients for analysis could vary from variable to variable and are reported separately for each analysis. For certain questions, survey logic was in place to prevent respondents from erroneous or contradictory data being entered.

Analysis was conducted in UNICOM Intelligence Reporter version 7.5 (UNICOM Systems, Inc.; 2021, Mission Hills, CA) [42].

### Ethical considerations

The DSP complies with all relevant guidelines and legal obligations in the countries where the survey was conducted, and in accordance with the Declaration of Helsinki (1964). Data were collected according to European Pharmaceutical Marketing Research Association guidelines and thus did not require ethics committee approvals [43]. The survey materials were submitted to the PEARL Institutional Review Board (REF: #22-ADRW-138) and following review were deemed to be exempt. The survey was performed in full accordance with relevant legislation at the time of data collection, including the US Health Insurance Portability and Accountability Act 1996 [44] and the Health Information Technology for Economic and Clinical Health Act legislation [45].

Using a checkbox, patients provided informed consent to take part in the survey. Data were collected in such a way that patients and physicians could not be identified directly. All data were anonymised and aggregated prior to receipt and analysis.

## Results

### Study sample

Overall, 74 neurologists participated in the physician survey, of which 53/74 were in EU5 (71.6%) and 21/74 in the US (28.4%). Neurologists reported caring for a median of 4.0 (Q1, Q3: 2.0; 6.0) patients with MOGAD in a typical month. Around half of neurologists had clinical trials experience in MOGAD at the time of the survey (currently involved: 5.4%, have been, but not currently involved: 47.3%, Table 1).

**Table 1:** Practice setting and clinical experience of participating neurologists who completed physician surveys, reported overall and by geography

	Overall	EU5	US
Number of MOGAD patients seen in a typical month			
Total number of respondents, *n*	74	53	21
Mean (SD)	5.2 (5.3)	5.6 (5.8)	4.1 (3.3)
Median (Q1, Q3)	4.0 (2.0; 6.0)	5.0 (2.0; 6.0)	3.0 (2.0; 5.0)
Min; max	1.0; 40.0	1.0; 40.0	1.0; 15.0
Clinical trial experience in MOGAD			
Total number of respondents, *n* (%)	74 (100)	53 (100)	21 (100)
Currently involved	4 (5.4)	4 (7.5)	0 (0)
Have been, but not currently involved	35 (47.3)	26 (49.1)	9 (42.9)
Never been involved	35 (47.3)	23 (43.4)	12 (57.1)

EU5, France, Germany, Italy, Spain, and United Kingdom; Q1, Q3, interquartile range; MOGAD, myelin-oligodendrocyte glycoprotein antibody-associated disease; *n*, number of physician surveys; SD, standard deviation

Neurologists completed PRFs for 268 patients with MOGAD, of which 213/268 were in EU5 (79.5%) and 55/268 in the US (20.5%). Overall, the median patient age at survey completion was 36.0 years (Q1, Q3: 28.0; 43.0 years), with a marginally younger population in EU5 compared to the US [median patient age 35.0 (Q1, Q3: 27.0; 43.0) and 38.0 (Q1, Q3: 35.0; 43.0), respectively]. In total, 173/268 (64.6%) of patients were female. Overall, most [229/268 (85.4%)] surveyed patients were White/Caucasian, with 195/213 (91.5%) in the EU5 sample and 34/55 (61.8%) in the US sample. Most of the patients, 171/268 (63.8%) did not have a physician-reported comorbidity as separate from their MOGAD diagnosis; among those who did have a comorbidity, the most prevalent was depression [39/97 (40.2%)]. The sample of patients included a higher proportion of monophasic patients [i.e., those who had not yet experienced a relapse at the time of data collection, 178/268 (66.4%)] than relapsing patients [90/268 (33.6%)] with similar characteristics (Table 2, Table S1).

**Table 2:** Patient demographics and clinical characteristics informed by participating neurologists, reported overall and by geography

	Overall	EU5	US
Age in years at survey completion			
Total number of patients, *n*	268	213	55
Mean (SD)	36.4 (11.1)	35.7 (11.3)	39.3 (9.6)
Median (Q1, Q3)	36.0 (28.0; 43.0)	35.0 (27.0; 43.0)	38.0 (35.0; 43.0)
Min; max	18.0; 75.0	18.0; 75.0	19; 62
Age at symptom onset in years			
Total number of patients with available information, *n*	223	180	43
Mean (SD)	32.8 (10.3)	31.9 (10.5)	36.5 (8.3)
Median (Q1, Q3)	32.5 (24.6; 39.7)	30.9 (23.7; 39.6)	36.8 (31.8; 40.7)
Min; max	12.5; 65.8	12.5; 65.8	19.0; 61.8
Age at definitive diagnosis in years			
Total number of patients with available information, *n*	236	187	49
Mean (SD)	33.6 (10.6)	32.4 (10.6)	38.1 (9.8)
Median (Q1, Q3)	33.3 (25.6; 40.1)	31.7 (24.0; 39.7)	37.4 (31.8; 42.4)
Min; max	15.6; 68.0	15.66; 68.0	19.0; 62.0
Sex			
Total number of patients, *n* (%)	268 (100)	213 (100)	55 (100)
Female	173 (64.6)	136 (63.8)	37 (67.3)
Male	95 (35.4)	77 (36.2)	18 (32.7)
Ethnicity^1^			
Total number of patients, *n* (%)	268 (100)	213 (100)	55 (100)
White/Caucasian	229 (85.4)	195 (91.5)	34 (61.8)
Hispanic/Latino	12 (4.5)	6 (2.8)	6 (10.9)
African American	11 (4.1)	0 (0.0)	11 (20.0)
Other	16 (6.0)	12 (5.7)	4 (7.3)
Comorbidities^2^, *n* (%)			
Total number of patients, *n* (%)	268 (100)	213 (100)	55 (100)
None reported	171 (63.8)	151 (70.9)	20 (36.4)
At least one reported^1^	97 (36.2)	62 (29.1)	35 (63.6)
Comorbidities among patients that reported at least one^1,2^			
Total number of patients, *n* (%)	97 (100)	62 (100)	35 (100)
Depression	39 (40.2)	25 (40.3)	14 (40.0)
Generalized anxiety	36 (37.1)	22 (35.4)	14 (40.0)
Diabetes without chronic complications	18 (18.6)	11 (17.7)	7 (< 1.0)
Disease duration			
Time since diagnosis at point of data collection, months	236	187	49
Median (IQR)	18.5 (8.9, 35.4)	19.3 (9.7, 40.0)	16.2 (7.9, 25.9)
Clinical course			
Total number of patients, *n* (%)	268 (100)	213 (100)	55 (100)
Monophasic	178 (66.4)	141 (66.2)	37 (67.3)
Relapsing	90 (33.6)	72 (33.8)	18 (32.7)

EU5, France, Germany, Italy, Spain, and United Kingdom; Q1, Q3, interquartile range; MOGAD, myelin-oligodendrocyte glycoprotein antibody-associated disease; *n*, number of PRF responses; PRF, patient record form; SD, standard deviation

^1^Categories are not mutually exclusive

^2^Diagnosed comorbidities as separate from MODAD diagnosis. Those with a prevalence in the total sample <2% not reported

In total, 66 patients (patient response rate: overall, 24.6%; EU5, 27.7%; US, 12.7%) completed PSCs, with no PSCs completed for the UK and Italy. Of the patients that completed the PSC questionnaire, 36/66 (54.5%) had a monophasic course and 30/66 (45.4%) had relapsing disease (Table S2).

### Diagnosis journey

The overall median age of patients at symptom onset was 32.5 years (Q1, Q3: 24.6; 39.7) and the median age at definitive diagnosis (positive serum MOG antibody test) was 33.3 years (Q1, Q3: 25.6; 40.1). Between regions, median age at symptom onset and at definitive diagnosis were greater in the US sample [36.8 (Q1, Q3: 31.8; 40.7) median years, and 37.4 (Q1, Q3: 31.8; 42.4) median years, respectively] than in the EU5 sample [30.9 (Q1, Q3: 23.7; 39.6) median years, and 31.7 (Q1, Q3: 24.0; 39.7) median years, respectively] (Table 2).

Prior to receiving a definitive MOGAD diagnosis, 64.2% of patients (172/268) were given preliminary or alternative diagnoses. Among patients who received a preliminary or alternative diagnoses, the three most frequent were optic neuritis [72/172 (41.9%)] multiple sclerosis [47/172 (27.3%)] and transverse myelitis [44/172 (25.6%)] (Fig. 2).

**Fig. 1:**
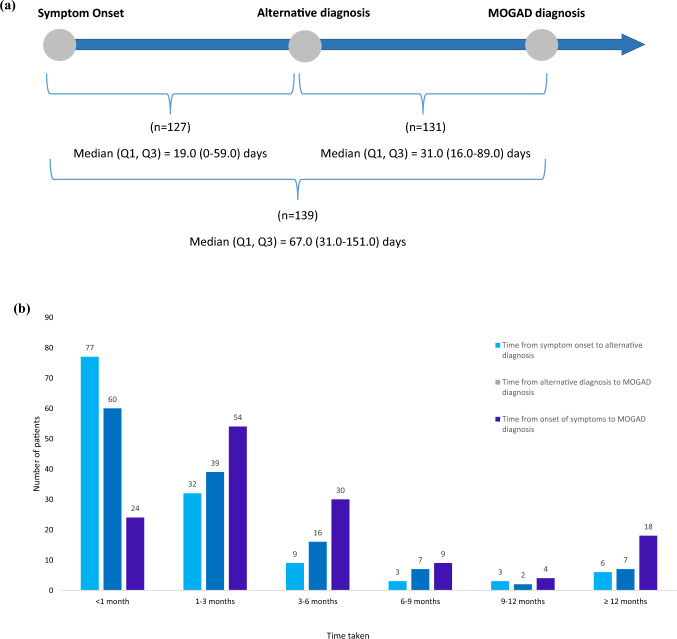
**a** Time from symptom onset to alternative diagnosis and MOGAD definitive diagnosis, among MOGAD diagnosed patients who received an alternative diagnosis prior to MOGAD (*n* = 172), informed by participating neurologists. Abbreviations: Q1, Q3, interquartile range; MOGAD, myelin-oligodendrocyte glycoprotein antibody-associated disease; *n*, number of PRF responses; PRF, patient record form. The dates of symptom onset, alternative diagnosis and MOGAD diagnosis were recorded separately in the PRF, and *unknown* was allowed as a response in each case; therefore, the final number of patients included in each time point measurement could differ. **b** Distribution of time from symptom onset to alternative diagnosis and MOGAD diagnosis among patients with an alternative diagnosis (*n* = 139)

**Fig. 2:**
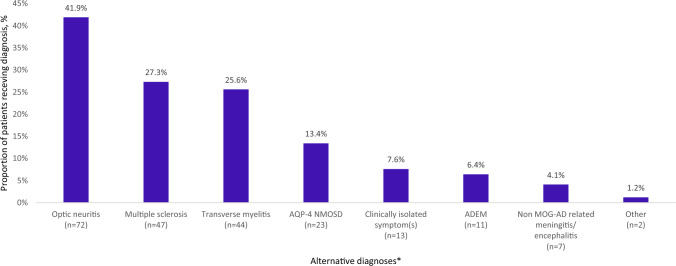
Alternative diagnoses before definitive MOGAD diagnosis, among MOGAD diagnosed patients who received an alternative diagnosis prior to MOGAD (*n* = 172), informed by participating neurologists. Abbreviations: ADEM, acute disseminated encephalomyelitis; AQP-4 NMOSD, aquaporin-4 neuromyelitis optica spectrum disorder; MOGAD, myelin-oligodendrocyte glycoprotein antibody-associated disease; *n*, number of PRF responses; PRF, patient record form. *Categories are not mutually exclusive

At the point of data collection, patients had been diagnosed with MOGAD a median (Q1, Q3) of 18.5 (8.3, 35.4) months. In EU5 this was a median (Q1, Q3) 19.3 (9.7, 40.0) months and in the US this was 16.2 (7.9, 25.9) months. When stratified by disease course, this was a median (IQR) 24.1 (14.7, 51.4) months in relapsing patients and 16.0 (6.7, 29.4) months in monophasic patients. Of those who received a preliminary or alternative diagnosis prior to definitive MOGAD diagnosis (*n* = 172/64.2%), the overall median (Q1, Q3) of physician-reported time from symptom onset to alternative diagnosis was 19.0 (0.0; 59.0) days, while the median (Q1, Q3) time from symptom onset to definitive diagnosis of MOGAD was 67.0 (31.0; 151.0) days. The overall median (Q1, Q3) time from alternative diagnosis to definitive diagnosis was 31.0 (16.0; 89.0) days (Fig. 1a). When looking at the frequency distribution of time from symptom onset to definitive diagnosis, 12% of patients had a diagnostic journey of 1 year or more (Fig. 1b). Among patients who received preliminary/alternative diagnoses, 96% (165/172) received acute treatment during their first symptomatic episode, compared with 84% (81/96) who didn’t receive a preliminary/alternative diagnosis (Fig. 2).

Overall, 50.8% of patients first consulted with a neurologist about their MOGAD symptoms, either the neurologist currently responsible for the patient’s care (91/268, 34.0%) or another neurologist (45/268, 16.8%). Signs prompting MOGAD antibody testing, where known, were most commonly negative serology for AQP-4 antibodies [165/268 (61.6%)], detection of longitudinally extensive myelitis [126/268 (47.0%)] and bilateral optic neuritis [116/268 (43.3%)]. The presence of extensive T2 and gadolinium (Gd)-enhancing lesion of the optic nerve or chiasm [60/268 (22.4%)], and perineural Gd-enhancement of the optic nerve upon MRI [53/268 (19.8%)], also prompted MOG antibody testing in ≥ 20% of patients in the overall sample. In 220/268 (82.1%) of cases, it was the neurologist currently responsible for the patient’s care who provided the patient with their diagnosis of MOGAD (Table 3).

**Table 3:** Patient first HCP interaction and signs prompting MOGAD antibody testing among patients diagnosed with MOGAD informed by participating neurologists, overall and by geography

	Overall	EU5	US
First HCP to consult with patient about their MOGAD symptoms			
Total number of patients, *n* (%)	268 (100)	213 (100)	55 (100)
Yourself (respondent)	91 (34.0)	78 (36.6)	13 (23.6)
Other neurologist	45 (16.8)	30 (14.1)	15 (27.3)
Ophthalmologist	29 (10.8)	23 (10.8)	6 (10.9)
Primary care physician (PCP)	50 (18.7)	40 (18.8)	10 (18.2)
Emergency care physician	36 (13.4)	29 (13.6)	7 (12.7)
Internist	6 (2.2)	3 (1.4)	3 (5.5)
Other	1 (< 1.0)	1 (< 1.0)	0 (0.0)
Don’t Know	10 (3.7)	9 (4.2)	1 (1.8)
Signs prompting MOGAD antibody testing			
Total number of patients, *n* (%)^1^	268 (100)	211 (100)	52 (100)
Negative serology for AQP-4 antibodies	165 (61.6)	133 (62.4)	32 (58.2)
Longitudinally extensive myelitis	126 (47.0)	102 (47.9)	24 (43.6)
Bilateral optic neuritis	116 (43.3)	92 (43.2)	24 (43.6)
Extensive T2 and Gd-enhancing lesion of optic nerve/chiasm	60 (22.4)	49 (23.0)	11 (20.0)
Perineural Gd-enhancement of optic nerve	53 (19.8)	41 (19.2)	12 (21.8)
MRI abnormalities confined to spinal cord gray matter	29 (10.8)	17 (8.0)	12 (21.8)
Conus medullaris involvement	24 (9.0)	20 (9.4)	4 (7.3)
ADEM	23 (8.6)	15 (7.0)	8 (14.5)
Other	9 (3.4)	6 (2.8)	3 (5.5)
Do not know	5 (1.9)	2 (0.9)	3 (5.5)
Healthcare professional that provided the patient with a diagnosis of MOGAD			
Total number of patients, *n* (%)	268 (100)	213 (100)	55 (100)
Yourself (respondent)	220 (82.1)	177 (83.1)	43 (78.2)
Other neurologist	42 (15.7)	34 (16.0)	8 (14.6)
Other	6 (2.2)	2 (< 1.0)	4 (7.3)

ADEM, acute disseminated encephalomyelitis; AQP-4, aquaporin-4; CSF, cerebrospinal fluid; CT, computed tomography; ELISA, enzyme-linked immunosorbent assay; EU5, France, Germany, Italy, Spain, and United Kingdom; HCP, healthcare professional; Gd, gadolinium; IgG, immunoglobulin G; LP, lumbar puncture; MOGAD, myelin-oligodendrocyte glycoprotein antibody-associated disease; MRI, magnetic resonance imaging; *n*, number of PRF responses; PCP, primary care physician; PRF, patient record form; SPEP, serum protein electrophoresis

^1^Categories are not mutually exclusive

To confirm MOGAD diagnosis, patients underwent a median 12.0 [Q1, Q3: 9.0; 19.0] assessments, tests and/or scans (see Table S3 for full list). The median number of assessments was 14.0 (Q1, Q3: 9.0; 20.0) in the EU5 sample and 9.0 (Q1, Q3: 5.0; 12.0) in the US sample. MOG antibody detection (cell-based assay) was used in all patients across regions to aid MOGAD diagnosis. Other frequently used tests (> 80% of all patients) included brain MRI (90.3%), spinal MRI (87.3%), and protein electrophoresis (81.7%). Additional assessments used to diagnose MOGAD in > 50% of all patients included physical examination (72.0%), complete blood count (62.7%), visual evoked potential (60.4%), visual field test (57.8%), visual acuity test (56.0%) and white cell count in the CSF (54.1%) Although the frequency of use for each test was generally lower in the US sample than in the EU5 sample, test ranking remained largely consistent across regions (Table S2).

### Reported symptoms

**Fig. 3:**
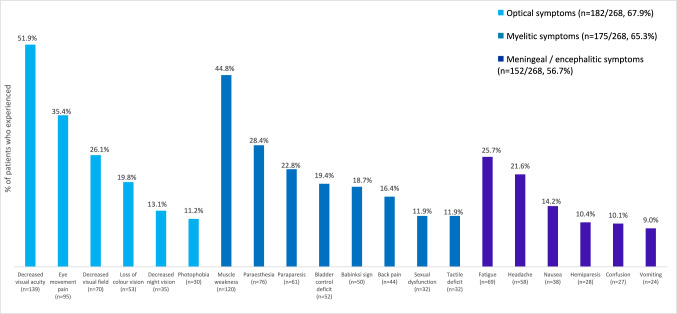
Initial presenting signs and symptoms among patients with MOGAD grouped by symptom category, informed by participating neurologists

Initial optic, myelitic, and/or meningeal/encephalitic presenting symptoms were present in substantial proportions of patients [182/268 (67.9%), 175/268 (65.3%) and 152/268 (56.7%) of patients, respectively]. The most common sign/symptoms across all categories were decreased visual acuity and muscle weakness, present in 139/268 (51.9%) and 120/268 (44.8%) of patients overall, respectively. Other signs/symptoms present in > 20% of patients included eye movement pain (35.4%), paraesthesia (28.4%), decreased visual field (26.2%), fatigue (25.7%), paraparesis (22.8%), and headache (21.6%) (Fig. 3).

The severity of initial presenting symptoms was wide ranging but generally moderate (Figure S1). Potentially disabling or serious signs were present in a limited number of patients with a prevalence < 10%. (i.e., cranial nerve palsy, unilateral blindness or bilateral blindness, seizures, respiratory failure, and coma).

### Clinical course and disease management

Overall, relapses were reported in 90/268 (33.6%) of patients. In patients with relapses, 77.8% of patients (70/90) were reported to have had only one relapse between the first symptomatic episode and survey completion. The median time from initial symptomatic episode to first relapse was 17.0 (Q1, Q3: 4.9; 29.0) months. Among patients with a relapse, symptoms were similarly distributed between optic, myelitic and meningeal/encephalitic symptoms (Table 4).

**Table 4:** Relapse characteristics of patients with relapsing MOGAD informed by participating neurologists, overall and by geography

	Overall	EU5	US
Total number of relapse episodes experienced among patients with at least one relapse			
Total number of patients with available information, *n*	90 (100)	72 (100)	18 (100)
1	70 (77.8)	55 (76.4)	15 (83.3)
2	14 (15.6)	12 (16.7)	2 (11.1)
3 +	6 (6.7)	5 (6.9)	1 (5.6)
Number of relapse episodes experienced among patients with at least one relapse			
Total number of patients with available information, *n*	90	72	18
Mean (SD)	1.3 (0.7)	1.3 (0.7)	1.3 (0.8)
Median (Q1, Q3)	1.0 (1.0; 1.0)	1.0 (1.0; 1.0)	1.0 (1.0; 1.0)
Min; max	1.0; 4.0	1.0; 4.0	1.0; 4.0
Time from initial symptomatic episode to first relapse (months) among patients with at least one relapse			
Total number of patients with available information, *n*	47	39	8
Mean (SD)	24.1 (31.5)	26.7 (33.9)	11.4 (7.9)
Median (Q1, Q3)	17.0 (4.9; 29.0)	20.0 (4.6; 32.0)	8.6 (5.8; 20.5)
Min; max	2.0; 179.0	2.0; 179.0	2.0; 23.0
Signs/symptoms during all relapse episodes among patients with at least one relapse			
Total number of patients, *n* (%)^1^	90 (100)	72 (100)	18 (100)
Optic	55 (62.5)	45 (64.3)	10 (55.7)
Myelitic	50 (56.8)	38 (54.3)	12 (66.7)
Meningeal/encephalitic	46 (52.3)	35 (50.0)	11 (61.1)
Do not know	2 (2.2)	2 (2.8)	0 (0.0)

EU5, France, Germany, Italy, Spain, and United Kingdom; Q1, Q3, interquartile range; MOGAD, myelin-oligodendrocyte glycoprotein antibody-associated disease; *n*, number of PRF responses; PRF, patient record form; SD, standard deviation

^1^Categories are not mutually exclusive

**Fig. 4:**
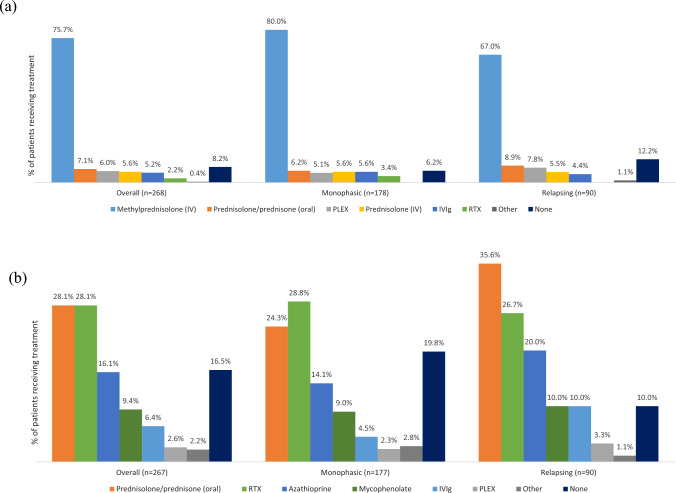
**a** Acute treatment received reported during the initial symptomatic episode among patients with MOGAD, informed by participating neurologists. **b** Maintenance treatment received after the initial symptomatic episode among patients with MOsGAD, informed by participating neurologists. Abbreviations: EU5, France, Germany, Italy, Spain, and United Kingdom; IV, intravenous; IVIg, IV immunoglobulins; MOGAD, MOGAD, myelin-oligodendrocyte glycoprotein antibody-associated disease; *n*, number of PRF responses; PLEX, plasma exchange; PRF, patient record form; RTX, rituximab

In total, 246/268 patients (91.8%) received treatment for an acute attack (i.e., provided to a patient at the first MOGAD attack). At the point of data collection, 79/246 (32%) of patients who received acute treatment were relapsing patients, compared to 167/246 (68%) of monophasic patients, with similar results also seen for patients receiving maintenance treatment [relapsing patients: 81/223 (36%) vs monophasic patients: 142/223 (64%)]. Of treatments received, the most frequently used was IV methylprednisolone, used in 203/268 (75.7%) of patients; more frequently among monophasic patients. Other treatments received by > 5% of patients included oral prednisolone/prednisone (7.1%), PLEX (6.0%), IV prednisolone (5.6%) and IVIg (5.2%) (Fig. 4a).

No maintenance treatment (long-term therapy aimed at preventing further MOGAD episodes) was prescribed to 44/267 (16.5%) of patients. The most common reasons given for this were that the patient refused medication [24/44 (54.6%)], that the patient was considered to have a very low risk of relapse [10/44 (22.7%)] and/or other not specified reason [8/44 (18.2%)] *(data not shown).* Maintenance treatment was received by 81/90 (90.0%) of relapsing patients compared to 142/177 (80.2%) of monophasic patients. Among patients who received maintenance treatment [223/267 (83.5%)], the most commonly received treatments were oral prednisolone/prednisone [75/267 (28.1%)] and rituximab [75/267 (28.1%)]. Other maintenance treatments received by < 20% of the patients included azathioprine (16.1%) mycophenolate (9.4%), IVIg (6.4%) and PLEX (2.6%) (Fig. 4b).

### Burden of disease

Hospitalisations over the last 12 months before survey completion were reported in 78/268 (29.1%) of patients overall. The median number of nights spent in hospital was 7.0 (Q1, Q3: 5.0; 9.0). The primary reason given for hospital admission was to receive plasmapheresis or infusion of treatment [38/78 (48.7%) of patients]. MOGAD relapses and complications were reasons for admission in 10/78 (12.8%) and 6/78 [7.7%] of patients, respectively. Where known, 48.7% of admissions (38/78) were not through the emergency room (ER), and 92.3% of patients (72/78) did not visit the intensive care unit (ICU) (Table 5).

**Table 5:** Hospitalization characteristics of patients with MOGAD in the last 12 months before survey completion informed by participating neurologists, reported overall and by clinical course

	Overall	Monophasic	Relapsing
Patients hospitalized in the last 12 months due to their MOGAD, *n* (%)	268 (100)	178 (100)	90 (100)
Yes	78 (29.1)	54 (30.3)	24 (26.7)
No	144 (53.7)	95 (53.4)	49 (54.4)
Do not know	46 (17.2)	29 (16.3)	17 (18.9)
Reason for admission of most recent hospitalization among patients with at least one hospitalization in the last 12 months			
Total number of patients, *n* (%)	78 (100)	54 (100)	24 (100)
To receive plasmapheresis or infusion of treatment	38 (48.7)	29 (53.7)	9 (37.5)
For a MOG-AD relapse	10 (12.8)	0 (0.0)	10 (41.6)
To treat a complication	6 (7.7)	5 (9.3)	1 (4.2)
For surgery	2 (2.6)	2 (3.7)	0 (0.0)
Other^1^	19 (24.4)	15 (27.8)	4 (16.7)
Do not know	3 (3.8)	3 (5.5)	0 (0.0)
Patient admitted through ER during most recent hospitalization among patients with at least one hospitalization in the last 12 months			
Total number of patients, *n* (%)	78 (100)	54 (100)	24 (100)
Yes	36 (46.2)	26 (48.1)	10 (41.6)
No	38 (48.7)	25 (46.3)	13 (54.2)
Do not know	4 (5.1)	3 (5.6)	1 (4.2)
Nights spent in hospital during most recent hospitalization among patients with at least 1 hospitalization in the last 12 months			
Total number of patients with available information, *n*	42	32	10
Mean (SD)	8.7 (7.3)	9.1 (7.5)	7.4 (6.5)
Median (Q1, Q3)	7.0 (5.0; 9.0)	7.0 (5.0; 10.5)	6.0 (4.0; 7.2)
Min; max	1.0; 33.0	1.0; 33.0	1.0; 25.0
Patient in ICU during most recent hospitalization among patients with at least one hospitalisation in the last 12 months			
Total number of patients with available information, *n*	78 (100)	54 (100)	24 (100)
Yes	5 (6.4)	4 (7.4)	1 (4.2)
No	72 (92.3)	49 (90.7)	23 (95.8)
Do not know	1 (1.3)	1 (1.9)	0 (0.0)

ER, emergency room; ICU, intensive care unit; Q1, Q3, interquartile range; MOGAD, myelin-oligodendrocyte glycoprotein antibody-associated disease; *n*, number of PRF responses; PLEX, plasma exchange; PRF, patient record form; SD, standard deviation

^1^Other reason is not captured

Neurologist-reported overall patient quality of life was most-frequently described as ‘very good’, ‘good’ or somewhat good’ [171/268 (63.8%) patients]. The extents to which MOGAD limited physical functioning, social functioning, and emotional wellbeing was most frequently described by neurologists as ‘a little’ [100/268 (37.3%), 94/268 (35.1%) and 92/268 (34.3%), respectively], followed by ‘a moderate amount’ [77/268 (28.7%), 68/268 (25.4%) and 67/268 (25.0%), respectively] (Table 6). As reported via the SF-36 questionnaire, patients most frequently self-reported that physical health/emotional problems interfered with normal social activities ‘not at all’ [26/66 (39.4%)] or ‘moderately’ [19/66 (28.8%)].

**Table 6:** Burden of disease related to MOGAD informed by participating neurologists and self-reported by patients with MOGAD, reported overall and by clinical course

	Overall	Monophasic	Relapsing
Reported by participating neurologists			
Overall quality of life			
Total number of patients, *n* (%)	268 (100)	178 (100)	90 (100)
Very poor	2 (< 1.0)	2 (1.1)	0 (0.0)
Poor	14 (5.2)	7 (3.9)	7 (7.8)
Somewhat poor	37 (13.8)	23 (12.9)	14 (15.6)
Neither poor nor good	36 (13.4)	23 (12.9)	13 (14.4)
Somewhat good	59 (22.0)	33 (18.5)	26 (28.9)
Good	67 (25.0)	49 (27.5)	18 (20.0)
Very good	45 (16.8)	36 (20.2)	9 (10.0)
Do not know	8 (3.0)	5 (2.8)	3 (3.3)
Extent to which MOGAD limited physical functioning			
Total number of patients, *n* (%)	268 (100)	178 (100)	90 (100)
Not at all	50 (18.7)	41 (23.1)	9 (10.0)
A little	100 (37.3)	70 (39.3)	30 (33.4)
A moderate amount	77 (28.7)	41 (23.0)	36 (40.0)
Substantially	27 (10.1)	16 (9.0)	11 (12.2)
Completely	11 (4.1)	8 (4.5)	3 (3.3)
Do not know	3 (1.1)	2 (1.1)	1 (1.1)
Extent to which MOGAD limited social functioning			
Total number of patients, *n* (%)	268 (100)	178 (100)	90 (100)
Not at all	65 (24.2)	50 (28.1)	15 (16.7)
A little	94 (35.1)	61 (34.3)	33 (36.7)
A moderate amount	68 (25.4)	41 (23.0)	27 (30.0)
Substantially	27 (10.1)	17 (9.5)	10 (11.1)
Completely	9 (3.3)	6 (3.4)	3 (3.3)
Do not know	5 (1.9)	3 (1.7)	2 (2.2)
Extent to which MOGAD affected emotional wellbeing			
Total number of patients, *n* (%)	268 (100)	178 (100)	90 (100)
Not at all	37 (13.8)	31 (17.4)	6 (6.7)
A little	92 (34.3)	63 (35.4)	29 (32.2)
A moderate amount	67 (25.0)	41 (23.0)	26 (28.9)
Substantially	52 (19.4)	31 (17.4)	21 (23.3)
Completely	13 (4.9)	8 (4.5)	5 (5.6)
Do not know	7 (2.6)	4 (2.3)	3 (3.3)
Self-reported by patients with MOGAD			
SF-36^1^: extent that physical health/emotional problems interfered with normal social activities			
Total number respondents, *n* (%)	66 (100)	36 (100)	30 (100)
Not at all	26 (39.4)	16 (44.4)	10 (33.4)
Slightly	15 (22.7)	9 (25.0)	6 (20.0)
Moderately	19 (28.8)	9 (25.0)	10 (33.0)
Quite a bit	5 (7.6)	2 (5.6)	3 (10.0)
Extremely	1 (1.5)	0 (0.0)	1 (3.3)

MOGAD, myelin-oligodendrocyte glycoprotein antibody-associated disease; *n*, number of responses; SF-36, Medical Outcomes Study 36-item Short-Form Health Survey

^1^The Medical Outcomes Study 36-item Short-Form Health Survey (SF-36) questionnaire is a generic survey used to measure self-reported Health-Related Quality of Life (HRQOL). It assesses various domains of physical and mental health, providing summary scores for physical and mental components

With respect to employment, 40.7% of patients were reported by physicians to be in full-time employment (109/268). Of those patients that were not in full-time employment (part-time work/on long-term sick leave/unemployed/retired due to MOGAD), 66.7% of these patients self-reported that this was due to reasons relating to MOGAD [monophasic patients (34/51 [66.7%]), relapsing patients (22/39 [56.4%]), with more missing responses among the relapsing patients]. In responses to the WPAI questionnaire, patients self-reported that the median of overall self-reported percentage of impairment while working, overall work impairment, and activity impairment due to MOGAD were 30.0% (Q1, Q3: 10.0%; 50.0%), 30.0% (Q1, Q3: 17.5%; 50.0%) and 30.0% (Q1, Q3: 10.0%; 50.0%), respectively (Table 7).

**Table 7:** Employment type and impairment among patients with MOGAD informed by participating neurologists and self-reported by patients with MOGAD, reported overall and by clinical course

	Overall	Monophasic	Relapsing
Reported by participating neurologists			
Employment status			
Total number of patients, *n* (%)	268 (100)	178 (100)	90 (100)
Full-time employment	109 (40.7)	77 (43.3)	32 (35.6)
Part-time employment	47 (17.5)	29 (16.3)	18 (20.0)
Student	45 (16.8)	35 (19.7)	10 (11.1)
Long-term sick leave	19 (7.1)	13 (7.3)	6 (6.7)
Unemployment	16 (6.0)	5 (2.8)	11 (12.2)
Homemaker	14 (5.2)	9 (5.0)	5 (5.6)
Retirement	8 (3.0)	4 (2.2)	4 (4.4)
Do not know	10 (3.7)	6 (3.4)	4 (4.4)
Part-time work/on long-term sick leave/unemployed/retired due to MOGAD			
Total number of patients, *n* (%)	90 (100)	51 (100)	39 (100)
Yes	56 (62.2)	34 (66.7)	22 (56.4)
No	28 (31.1)	16 (31.4)	12 (30.8)
Do not know	6 (6.7)	1 (1.9)	5 (12.8)
Self-reported by patients with MOGAD			
WPAI ^1^: percentage work time missed due to problem			
Total number of respondents, *n*	36	33	3
Mean (SD)	6.9 (23.3)	7.5 (24.3)	0.0 (0.0)
Median (Q1, Q3)	(0.0 (0.0; 0.0)	(0.0 (0.0; 0.0)	0.0 (0.0; 0.0)
Min; max	0.0; 100.0	0.0; 100.0	0.0; 0.0
WPAI ^1^: percentage impairment while working due to problem			
Total number of respondents, *n*	35	16	19
Mean (SD)	30.3 (21.7)	25.6 (22.2)	34.2 (19.8)
Median (Q1, Q3)	30.0 (10.0; 50.0)	20.0 (2.5; 50.0)	30.0 (20.0; 50.0)
Min; max	0.0; 80.0	0.0; 60.0	0.0; 80.0
WPAI ^1^: percentage overall work impairment due to problem			
Total number of respondents, *n*	34	16	18
Mean (SD)	31.4 (21.8)	26.3 (23.1)	35.9 (20.1)
Median (Q1, Q3)	30.0 (17.5; 50.0)	20.0 (2.5; 50.0)	35.0 (27.5; 50.0)
Min; max	0.0; 85.0	0.0; 64.0	0.0; 85.0
WPAI ^1^: percentage activity impairment due to problem			
Total number of respondents, *n*	66	36	30
Mean (SD)	35.0 (27.9)	29.4 (29.9)	41.7 (24.1)
Median (Q1, Q3)	30.0 (10.0; 50.0)	20.0 (0.0; 47.5)	40.0 (27.5; 60.0)
Min; max	0.0; 100.0	0.0; 100.0	s0.0; 100.0

EU5, France, Germany, Italy, Spain, and United Kingdom; IQR, interquartile range; MOGAD, myelin-oligodendrocyte glycoprotein antibody-associated disease; *n*, number of responses; SD, standard deviation; WPAI, Work Productivity and Activity Impairment Questionnaire

^1^The Work Productivity and Activity Impairment (WPAI) questionnaire measures the effect of a health problem on work productivity and regular activities. The WPAI uses six questions to measure patient-reported absenteeism, presenteeism, and daily activity impairment attributed to the disease, quantified as a percentage impairment

## Discussion

This study, conducted across Europe and the US, aimed to generate insights on the disease management and burden of MOGAD using real-world survey data from neurologists and their adult MOGAD patients. Overall, the population of patients sampled was broadly consistent in terms of demographic and clinical characteristics with populations in previously published studies [6, 46]. MOGAD often presents in childhood. While the original survey did collect data for a small number of pediatric patients (*n* = 39), these patients were excluded from the study to not complicate the primary analysis. There is evidence in literature that MOGAD pediatric patients have a different presentation of disease compared to adults [8, 47].

Among patients that were reported to have at least one comorbidity, a significant proportion experienced depression, in alignment with other recent studies [18, 19]. Approximately one-third of patients included in the present cross-sectional study were reported to have a relapsing clinical course, however we did not collect data on relapse duration or follow-up duration post relapse. Due to the cross-sectional study design, patients were at different points in their management journey, and follow-up data were not collected. When stratified by disease course, at the point of data collection, the median (IQR) time since diagnosis was 24.1 (14.7, 51.4) months in relapsing patients and 16.0 (6.7, 29.4) months in monophasic patients. Accordingly, some patients classified as monophasic at the point of data collection may transition to a relapsing disease course, and thus the proportion of patients classified as monophasic in this sample may be overrepresented. This proportion of relapsing patients was lower compared with previous published literature: in two other studies in adult populations, relapse rates were 42% and 58% [6, 46]. Cobo-Calvo and colleagues showed in their study that the probability of relapsing increased over time, this was 45% 2 years and 62% after 5 years [6]. In the study by Jarius and colleagues, relapse rate was reported as 80% over a mean (SD) time since disease onset of 75 (46.5) months [48].

Prompt diagnosis is vital, as the prognosis and treatment of MOGAD is different to both MS and NMOSD [2, 3, 20]. The current survey found evidence of challenges in the diagnosis of MOGAD, as 64.2% of patients received preliminary/alternative diagnoses before being definitively diagnosed. It could be inferred that the alternative diagnosis is used as a suspected preliminary diagnosis before the definitive one, particularly as half of the patients initially consulted with a physician with a specialty other than neurologist at symptom onset (Table 3). Nonetheless, the physician-reported median time between symptom onset and alternative diagnosis in this study was notably short compared to other studies [49], which may require further exploration. In a recent international real-world treatment pathway survey, data from patients with MOGAD or their caregivers indicated diagnostic delays [49]. Results from 204 patients/caregivers showed that 18% of patients experienced a diagnostic delay of at least 5 years, and 55% received an alternative diagnosis, before definitive MOGAD diagnosis [49]. One avenue for future work would be an analysis of factors which influenced diagnostic delay, and the impact on treatment choices and outcomes. In the present study, while overall physician-reported median time to diagnosis was short, 12% of patients experienced diagnostic delays of 1 year or more from symptom onset to definitive diagnosis. In those patients diagnosed prior to widespread antibody testing, the diagnosis pathway would likely have been longer.

The patients in this sample were typically diagnosed with MOGAD by a neurologist following a median of 12 assessments, tests and/or scans, likely increasing the burden to the individual and the healthcare system. The recently proposed 2023 Banwell diagnostic criteria suggest that the presence of MOG antibodies, and clinical and MRI evidence are required for a confirmatory MOGAD diagnosis, once potential alternatives such as multiple sclerosis have been excluded [15]. At the time the original survey was conducted, the 2023 Banwell criteria were not available, and due to the cross-sectional nature of data collection these criteria could not be applied to individual patients retrospectively in this study. With the recent introduction of the diagnosis code for MOGAD in the ICD-10-CM in the US, it is likely that the diagnosis pathway for MOGAD will continue to change [24] and improve diagnostic journey in future.

In the current study, it was shown that the treatment of MOGAD patients, in both acute and maintenance settings, remains corticosteroid based. This aligns with prior surveys of the treatment landscape for MOGAD, with corticosteroids being favored for acute treatment and to reduce relapse rates as maintenance therapy [3, 26, 50]. However, long-term use of corticosteroids are known to result in an increasing likelihood of side effects [51]. As acute treatment, most patients received IV methylprednisolone (75.7%), with few patients receiving other treatment options. As maintenance treatment, after corticosteroids, rituximab was the second most common treatment observed in the present study both in monophasic (28.8%) and relapsing patients (26.7%), despite a lack of randomized control trial evidence supporting its use in MOGAD [52]. From retrospective evidence, IVIg appears more effective in reducing relapse rates than mycophenolate mofetil, rituximab, azathioprine, or prednisone [53, 54] and long-term maintenance treatments with better benefit–risk profiles are still an unmet need for patients with MOGAD. Irrespective of disease course, a high number of patients in this study were receiving both acute and maintenance treatments, which may reflect difficulties in predicting disease course in individual patients and the lack of consensus when to start preventive immunotherapy [9, 30].

Although 66.4% of patients in this study were reported to have monophasic disease, i.e., had not yet experienced a relapse at the time of data collection, it was notable that maintenance treatment was not pursued in 16.5% of the overall sample. One caveat to this is that given the cross-sectional design, patients were at different points in the management journey. The most common reason for not receiving maintenance treatment in more than half of these patients was refusal, followed by being considered at low risk of relapse. The detailed reasons for requesting no medication were not captured, and predictive factors for a relapsing disease course require further investigations [9]. As disability worsening is associated with relapses [55], patient education regarding the importance of maintenance treatment in preventing relapses is crucial.

Burden of disease data on hospitalizations showed that 29.1% of patients were hospitalized in the last 12 months before the survey. The most commonly reported reason for hospitalization was to receive plasmapheresis or treatment infusions (48.7%), in alignment with the high proportion of patients reported to receive IV methylprednisolone as acute treatment (75.7%. Patient quality of life was commonly reported by neurologists as ‘very good’, ‘good’, or ‘somewhat good’ (63.8%), with better ratings for monophasic than relapsing patients. Similarly, based on the SF-36 questionnaire, patients who had at least one relapse reported most frequently poorer quality of life than those who were monophasic, which may highlight the need for better treatment options for the relapsing population. In this study, among patients with a relapse the symptoms were similarly distributed between optic, myelitic and meningeal/encephalitic symptoms. This differs to other studies which suggest that at relapse optic symptoms are more frequent [5, 11].

There was a non-negligible level of work-related impairment. Physicians reported less than half of patients to be in full-time employment, with MOGAD was reported to be the cause for 62% of patients who were not working full-time. This is in agreement with a previous study in a cohort of patients with MOGAD or NMOSD which found that 60% of the cohort was unemployed, and that a third of these had retired as a result of their disease [32]. From patient self-reported data, the median work impartment percentage among patients who were working was around one third, with higher proportions of work impairment also observed in the group of relapsing patients.

### Limitations

This was a cross-sectional study, with neurologists completing PRFs for consecutively consulting patients with MOGAD. Only patients consulting with their treating neurologists were included in the study, and it is therefore recognized that the sample is more likely to include patients who were visiting neurologists more often, likely presenting different demographic or clinical profiles such as severity status and clinical course compared with the wider population of patients with MOGAD, which may have introduced selection bias.

Conversely, patients with more severe disease activity may have declined to complete the voluntary questionnaire, which may account for the low completion rate seen in this study. Participants were encouraged, but not mandated, to complete all questions voluntarily, where social desirability bias might have played a role [56]. As a result, sample size bases for each question fluctuated across different domains leading to incomplete data collection. In particular, sample sizes for the US patients were low for some questions included, limiting the conclusions that could be drawn.

It should be noted that the survey was designed to facilitate understanding of real-world clinical practice, and thus neurologists could presumably only report on data they had to hand at the time of the consultation. Therefore, the study represents the evidence they had when making any clinical treatment and other management decisions at that consultation. No tests, treatments, or other investigations were performed as part of this survey.

Finally, the study relies on the accuracy of neurologists when completing each PRF and the willingness of patients to complete their PSCs, which were voluntary. Some information in the survey may be subject to recall bias. To minimize this risk, data were encouraged to be collected at the time of each patient’s appointment, and physicians had access to patient medical records for extraction of retrospective data. In addition, the questionnaires were relatively short and user-friendly with electronic routing and logic applied to ensure no contradictions in responses.

## Conclusion

This real-world study suggests that patients with MOGAD may suffer from challenges in prompt diagnosis despite undergoing numerous tests and scans. MOGAD management itself remains suboptimal, with burden to patients reflected in their quality of life and ability to work. Both the diagnosis and treatment of MOGAD should continue to be the subject of further research. The generation of robust data is vital to ultimately aid the optimization of the available treatment options. The optimization of diagnosis protocols and treatment guidelines should also continue.

## Supplementary Information

Below is the link to the electronic supplementary material.

Supplementary file1 (DOCX 78 KB)

## Data Availability

All data, i.e., methodology, materials, data and data analysis, that support the findings of this survey are the intellectual property of Adelphi Real World. All reasonable requests for access should be addressed directly to Mia Unsworth at mia.unsworth@adelphigroup.com.

## References

[1] Marignier R, Hacohen Y, Cobo-Calvo A, Pröbstel AK, Aktas O, Alexopoulos H et al (2021) Myelin-oligodendrocyte glycoprotein antibody-associated disease. Lancet Neurol 20(9):762–772 34418402 10.1016/S1474-4422(21)00218-0

[2] Sechi E, Cacciaguerra L, Chen JJ, Mariotto S, Fadda G, Dinoto A et al (2022) Myelin oligodendrocyte glycoprotein antibody-associated disease (MOGAD): a review of clinical and MRI features, diagnosis, and management. Front Neurol 13:885218 35785363 10.3389/fneur.2022.885218 PMC9247462

[3] Al-Ani A, Chen JJ, Costello F (2023) Myelin oligodendrocyte glycoprotein antibody-associated disease (MOGAD): current understanding and challenges. J Neurol 270(8):4132–4150 37154894 10.1007/s00415-023-11737-8 PMC10165591

[4] Hor JY, Fujihara K (2023) Epidemiology of myelin oligodendrocyte glycoprotein antibody-associated disease: a review of prevalence and incidence worldwide. Front Neurol 14:1260358 37789888 10.3389/fneur.2023.1260358 PMC10542411

[5] Jurynczyk M, Messina S, Woodhall MR, Raza N, Everett R, Roca-Fernandez A et al (2017) Clinical presentation and prognosis in MOG-antibody disease: a UK study. Brain 140(12):3128–3138 29136091 10.1093/brain/awx276

[6] Cobo-Calvo A, Ruiz A, Maillart E, Audoin B, Zephir H, Bourre B et al (2018) Clinical spectrum and prognostic value of CNS MOG autoimmunity in adults: the MOGADOR study. Neurology 90(21):e1858–e1869 29695592 10.1212/WNL.0000000000005560

[7] Waters P, Fadda G, Woodhall M, O’Mahony J, Brown RA, Castro DA et al (2020) Serial anti-myelin oligodendrocyte glycoprotein antibody analyses and outcomes in children with demyelinating syndromes. JAMA Neurol 77(1):82–93 31545352 10.1001/jamaneurol.2019.2940 PMC6763982

[8] Cobo-Calvo A, Ruiz A, Rollot F, Arrambide G, Deschamps R, Maillart E et al (2021) Clinical features and risk of relapse in children and adults with myelin oligodendrocyte glycoprotein antibody-associated disease. Ann Neurol 89(1):30–41 32959427 10.1002/ana.25909

[9] Chen B, Gomez-Figueroa E, Redenbaugh V, Francis A, Satukijchai C, Wu Y et al (2023) Do early relapses predict the risk of long-term relapsing disease in an adult and paediatric cohort with MOGAD? Ann Neurol 94(3):508–517 37394961 10.1002/ana.26731

[10] Duchow A, Bellmann-Strobl J, Friede T, Aktas O, Angstwurm K, Ayzenberg I et al (2024) Time to disability milestones and annualized relapse rates in NMOSD and MOGAD. Ann Neurol 95(4):720–732 38086777 10.1002/ana.26858

[11] López-Chiriboga AS, Majed M, Fryer J, Dubey D, McKeon A, Flanagan EP et al (2018) Association of MOG-IgG serostatus with relapse after acute disseminated encephalomyelitis and proposed diagnostic criteria for MOG-IgG-associated disorders. JAMA Neurol 75(11):1355–1363 30014148 10.1001/jamaneurol.2018.1814 PMC6248120

[12] Armangue T, Olivé-Cirera G, Martínez-Hernandez E, Sepulveda M, Ruiz-Garcia R, Muñoz-Batista M et al (2020) Associations of paediatric demyelinating and encephalitic syndromes with myelin oligodendrocyte glycoprotein antibodies: a multicentre observational study. Lancet Neurol 19(3):234–246 32057303 10.1016/S1474-4422(19)30488-0

[13] Akaishi T, Misu T, Fujihara K, Takahashi T, Takai Y, Nishiyama S et al (2022) Relapse activity in the chronic phase of anti-myelin-oligodendrocyte glycoprotein antibody-associated disease. J Neurol 269(6):3136–3146 34820735 10.1007/s00415-021-10914-x PMC9120114

[14] Satukijchai C, Mariano R, Messina S, Sa M, Woodhall MR, Robertson NP et al (2022) Factors associated with relapse and treatment of myelin oligodendrocyte glycoprotein antibody-associated disease in the United Kingdom. JAMA Netw Open 5(1):e2142780 35006246 10.1001/jamanetworkopen.2021.42780 PMC8749481

[15] Banwell B, Bennett JL, Marignier R, Kim HJ, Brilot F, Flanagan EP et al (2023) Diagnosis of myelin oligodendrocyte glycoprotein antibody-associated disease: International MOGAD Panel proposed criteria. Lancet Neurol 22(3):268–282 36706773 10.1016/S1474-4422(22)00431-8

[16] Fabri TL, O’Mahony J, Fadda G, Gur RE, Gur RC, Yeh EA et al (2022) Cognitive function in pediatric-onset relapsing myelin oligodendrocyte glycoprotein antibody-associated disease (MOGAD). Mult Scler Relat Disord 59:103689 35183817 10.1016/j.msard.2022.103689

[17] Kazzi C, Alpitsis R, O’Brien TJ, Malpas CB, Monif M (2024) Cognitive and psychopathological features of neuromyelitis optica spectrum disorder and myelin oligodendrocyte glycoprotein antibody-associated disease: a narrative review. Mult Scler Relat Disord 85:105596 38574722 10.1016/j.msard.2024.105596

[18] Asseyer S, Henke E, Trebst C, Hümmert MW, Wildemann B, Jarius S et al (2021) Pain, depression, and quality of life in adults with MOG-antibody–associated disease. Eur J Neurol 28(5):1645–1658 33423336 10.1111/ene.14729

[19] Said Y, Ladakis DC, Lefelar JM, Khazen JM, Gould J, Fitzgerald KC et al (2024) Quality of life is impaired in myelin oligodendrocyte glycoprotein antibody associated disease. Mult Scler J Exp Transl Clin 10(3):20552173241274604 39185445 10.1177/20552173241274605 PMC11342330

[20] Bartels F, Lu A, Oertel FC, Finke C, Paul F, Chien C (2021) Clinical and neuroimaging findings in MOGAD-MRI and OCT. Clin Exp Immunol 206(3):266–281 34152000 10.1111/cei.13641 PMC8561692

[21] Lennon VA, Wingerchuk DM, Kryzer TJ, Pittock SJ, Lucchinetti CF, Fujihara K et al (2004) A serum autoantibody marker of neuromyelitis optica: distinction from multiple sclerosis. Lancet 364(9451):2106–2112 15589308 10.1016/S0140-6736(04)17551-X

[22] O’Connor KC, McLaughlin KA, De Jager PL, Chitnis T, Bettelli E, Xu C et al (2007) Self-antigen tetramers discriminate between myelin autoantibodies to native or denatured protein. Nat Med 13(2):211–217 17237795 10.1038/nm1488 PMC3429369

[23] Reindl M, Waters P (2019) Myelin oligodendrocyte glycoprotein antibodies in neurological disease. Nat Rev Neurol 15(2):89–102 30559466 10.1038/s41582-018-0112-x

[24] ICD10Data. ICD-10-CM Diagnosis Code G37.81. 2024 2024 [21 June 2024]. Available from: https://www.icd10data.com/ICD10CM/Codes/G00-G99/G35-G37/G37-/G37.81#:~:text=Myelin%20oligodendrocyte%20glycoprotein%20antibody%20disease,-2024%20%2D%20New%20Code&text=81%20is%20a%20billable%2Fspecific,10%2DCM%20version%20of%20G37.

[25] Klein da Costa B, Banwell BL, Sato DK (2021) Treatment of MOG-IgG associated disease in paediatric patients: a systematic review. Mult Scler Relat Disord 56:103216 34450460 10.1016/j.msard.2021.103216

[26] Cacciaguerra L, Flanagan EP (2024) Updates in NMOSD and MOGAD diagnosis and treatment: a tale of two central nervous system autoimmune inflammatory disorders. Neurol Clin 42(1):77–114 37980124 10.1016/j.ncl.2023.06.009 PMC10658081

[27] Ringelstein M, Ayzenberg I, Lindenblatt G, Fischer K, Gahlen A, Novi G et al (2022) Interleukin-6 receptor blockade in treatment-refractory MOG-IgG-associated disease and neuromyelitis optica spectrum disorders. Neurol Neuroimmunol Neuroinflamm 9(1):e1100 34785575 10.1212/NXI.0000000000001100 PMC8596357

[28] Chen JJ, Huda S, Hacohen Y, Levy M, Lotan I, Wilf-Yarkoni A et al (2022) Association of maintenance intravenous immunoglobulin with prevention of relapse in adult myelin oligodendrocyte glycoprotein antibody-associated disease. JAMA Neurol 79(5):518–525 35377395 10.1001/jamaneurol.2022.0489 PMC8981066

[29] Carnero Contentti E, Marrodan M, Correale J (2021) Emerging drugs for the treatment of adult MOG-IgG-associated diseases. Expert Opin Emerg Drugs 26(2):75–78 33861167 10.1080/14728214.2021.1919082

[30] Whittam DH, Karthikeayan V, Gibbons E, Kneen R, Chandratre S, Ciccarelli O et al (2020) Treatment of MOG antibody associated disorders: results of an international survey. J Neurol 267(12):3565–3577 32623595 10.1007/s00415-020-10026-y PMC7954658

[31] Wynford-Thomas R, Jacob A, Tomassini V (2019) Neurological update: MOG antibody disease. J Neurol 266(5):1280–1286 30569382 10.1007/s00415-018-9122-2 PMC6469662

[32] Hümmert MW, Schöppe LM, Bellmann-Strobl J, Siebert N, Paul F, Duchow A et al (2022) Costs and health-related quality of life in patients with NMO Spectrum disorders and MOG-antibody-associated disease: CHANCE(NMO) study. Neurology 98(11):e1184–e1196 35082170 10.1212/WNL.0000000000200052 PMC8935443

[33] Sarnes E, Crofford L, Watson M, Dennis G, Kan H, Bass D (2011) Incidence and US costs of corticosteroid-associated adverse events: a systematic literature review. Clin Ther 33(10):1413–1432 21999885 10.1016/j.clinthera.2011.09.009

[34] Anderson P, Benford M, Harris N, Karavali M, Piercy J (2008) Real-world physician and patient behaviour across countries: disease-specific programmes—a means to understand. Curr Med Res Opin 24(11):3063–3072 18826746 10.1185/03007990802457040

[35] Anderson P, Higgins V, Courcy J, Doslikova K, Davis VA, Karavali M et al (2023) Real-world evidence generation from patients, their caregivers and physicians supporting clinical, regulatory and guideline decisions: an update on Disease Specific Programmes. Curr Med Res Opin 39(12):1707–1715 37933204 10.1080/03007995.2023.2279679

[36] Babineaux SM, Curtis B, Holbrook T, Milligan G, Piercy J (2016) Evidence for validity of a national physician and patient-reported, cross-sectional survey in China and UK: the Disease Specific Programme. BMJ Open 6(8):e010352 27531722 10.1136/bmjopen-2015-010352 PMC5013497

[37] Higgins V, Piercy J, Roughley A, Milligan G, Leith A, Siddall J et al (2016) Trends in medication use in patients with type 2 diabetes mellitus: a long-term view of real-world treatment between 2000 and 2015. Diabetes Metab Syndr Obes 9:371–380 27843332 10.2147/DMSO.S120101 PMC5098530

[38] Reilly MC, Zbrozek AS, Dukes EM (1993) The validity and reproducibility of a work productivity and activity impairment instrument. Pharmacoeconomics 4(5):353–365 10146874 10.2165/00019053-199304050-00006

[39] McHorney CA, Ware JE Jr, Raczek AE (1993) The MOS 36-Item Short-Form Health Survey (SF-36): II. Psychometric and clinical tests of validity in measuring physical and mental health constructs. Med Care 31(3):247–263 8450681 10.1097/00005650-199303000-00006

[40] McHorney CA, Ware JEJ, Rachel Lu JF, Sherbourne CD (1994) The MOS 36-ltem Short-Form Health Survey (SF-36): III. Tests of data quality, scaling assumptions, and reliability across diverse patient groups. Med Care 32(1):40–66 8277801 10.1097/00005650-199401000-00004

[41] Ware JE Jr, Sherbourne CD (1992) The MOS 36-item short-form health survey (SF-36). I. Conceptual framework and item selection. Med Care 30(6):473–483 1593914

[42] UNICOM Systems I. Intelligence Reporter version 7.5. Mission Hills, CA: UNICCOM Systems, Inc.; 2021.

[43] European Pharmaceutical Market Research Association (EphMRA). Code of Conduct 2023 updated September 2023. Available from: https://www.ephmra.org/sites/default/files/2023-09/2023%20EPHMRA%20Code%20of%20Conduct%2025.9.23.pdf.

[44] US Department of Health and Human Services. Summary of the HIPAA Privacy Rule 2003. Available from: http://www.hhs.gov/sites/default/files/privacysummary.pdf.

[45] Health Information Technology (HITECH). Health Information Technology Act 2009. Available from: https://www.healthit.gov/sites/default/files/hitech_act_excerpt_from_arra_with_index.pdf.

[46] Sutton P, Lutz MW, Hartsell FL, Kimbrough D, Tagg NT, Skeen M et al (2022) Myelin oligodendrocyte glycoprotein (MOG) antibody-associated disease: presentation and outcomes of adults at a single center. J Neuroimmunol 373:577987 36272183 10.1016/j.jneuroim.2022.577987

[47] Trewin BP, Brilot F, Reddel SW, Dale RC, Ramanathan S (2025) MOGAD: a comprehensive review of clinicoradiological features, therapy and outcomes in 4699 patients globally. Autoimmun Rev 24(1):103693 39577549 10.1016/j.autrev.2024.103693

[48] Jarius S, Ruprecht K, Kleiter I, Borisow N, Asgari N, Pitarokoili K et al (2016) MOG-IgG in NMO and related disorders: a multicenter study of 50 patients. Part 2: epidemiology, clinical presentation, radiological and laboratory features, treatment responses, and long-term outcome. J Neuroinflammation 13(1):280 27793206 10.1186/s12974-016-0718-0 PMC5086042

[49] Santoro JD, Gould J, Panahloo Z, Thompson E, Lefelar J, Palace J (2023) Patient Pathway to diagnosis of myelin oligodendrocyte glycoprotein antibody-associated disease (MOGAD): findings from a multinational survey of 204 patients. Neurol Ther 12(4):1081–1101 37024731 10.1007/s40120-023-00474-9 PMC10310677

[50] Chwalisz BK, Levy M (2022) The treatment of myelin oligodendrocyte glycoprotein antibody disease: a state-of-the-art review. J Neuroophthalmol 42(3):292–296 35944137 10.1097/WNO.0000000000001684

[51] Buchman AL (2001) Side effects of corticosteroid therapy. J Clin Gastroenterol 33(4):289–294 11588541 10.1097/00004836-200110000-00006

[52] Spagni G, Sun B, Monte G, Sechi E, Iorio R, Evoli A et al (2023) Efficacy and safety of rituximab in myelin oligodendrocyte glycoprotein antibody-associated disorders compared with neuromyelitis optica spectrum disorder: a systematic review and meta-analysis. J Neurol Neurosurg Psychiatry 94(1):62–69 36283808 10.1136/jnnp-2022-330086

[53] Chen JJ, Flanagan EP, Bhatti MT, Jitprapaikulsan J, Dubey D, Lopez Chiriboga ASS et al (2020) Steroid-sparing maintenance immunotherapy for MOG-IgG associated disorder. Neurology 95(2):e111–e120 32554760 10.1212/WNL.0000000000009758 PMC7455322

[54] Bilodeau PA, Vishnevetsky A, Molazadeh N, Lotan I, Anderson M, Romanow G et al (2024) Effectiveness of immunotherapies in relapsing myelin oligodendrocyte glycoprotein antibody-associated disease. Mult Scler 30(3):357–368 38314479 10.1177/13524585241226830

[55] Akaishi T, Misu T, Takahashi T, Takai Y, Nishiyama S, Fujimori J et al (2021) Progression pattern of neurological disability with respect to clinical attacks in anti-MOG antibody-associated disorders. J Neuroimmunol 351:577467 33388541 10.1016/j.jneuroim.2020.577467

[56] Bispo Júnior JP (2022) Social desirability bias in qualitative health research. Rev Saude Publica 56:101 36515303 10.11606/s1518-8787.2022056004164 PMC9749714

