# Supplementary material for: Management and burden of disease in people with myelin oligodendrocyte glycoprotein antibody-associated disease: data from an international, cross-sectional survey
Source: J Neurol. 2025 Jul 22;272(8):529. doi: 10.1007/s00415-025-13233-7 (PMC12283468; doi:10.1007/s00415-025-13233-7)
Supplement: Supplementary file 1 — Supplementary file1 (DOCX 78 KB) [file 415_2025_13233_MOESM1_ESM.docx]

**Table S1. Patient demographics and clinical characteristics informed by participating neurologists, overall and by clinical course**

|  | **Overall** | **Monophasic** | **Relapsing** |
| --- | --- | --- | --- |
| **Age in years at survey completion** |  |  |  |
| **Total number of patients, n**  Mean (SD)  Median (Q1,Q2)  Min; max | **268**  36.4 (11.1)  36.0 (28.0; 43.0)  18.0; 75.0 | **178**  35.6 (10.9)  35.0 (27.8; 42.0)  18.0; 75.0 | **90**  38.0 (11.3)  38.0 (30.0; 45.0)  18.0; 73.0 |
| **Age in years at diagnosis** |  |  |  |
| **Total number of patients, n**  Mean (SD)  Median (Q1,Q2)  Min; max | **236**  33.6 (10.6)  33.3 (25.6; 40.1)  16.0; 68.0 | **161**  33.5 (10.4)  33.8 (25.2; 40.3)  16.0; 64.0 | **75**  33.8 (11.1)  32.8 (26.5; 39.8)  17.0; 68.0 |
| **Age in years at symptom onset** |  |  |  |
| **Total number of patients, n**  Mean (SD)  Median (Q1,Q2)  Min; max | **223**  32.8 (10.2)  32.5 (24.6; 39.7)  12.0; 66.0 | **155**  33.0 (10.3)  32.5 (24.6; 40.2)  16.0; 64.0 | **68**  32.3 (10.3)  32.5 (25.0; 38.9)  17.0; 68.0 |
| **Sex** |  |  |  |
| **Total number of patients, n (%)**  Female Male | **268 (100)**  173 (64.6) 95 (35.4) | **178 (100)**  115 (64.6) 63 (35.4) | **90 (100)**  58 (64.4) 32 (35.6) |
| **Ethnicity^1^** |  |  |  |
| **Total number of patients, n (%)**  White/Caucasian  Hispanic/Latino  African American  Other | **268 (100)**  229 (85.4)  12 (4.5)  11 (4.1)  16 (6.0) | **178 (100**  148 (83.1)  9 (5.1)  7 (3.9)  14 (7.9) | **90 (100)**  81 (90.0)  3 (3.3)  4 (4.4)  2 (2.2) |
| **Comorbidities** |  |  |  |
| **Total number of patients, n (%)**  None reported  At least one reported | **268 (100)**  171 (63.8)  97 (36.2) | **178 (100)**  118 (66.3)  60 (33.7) | **90 (100)**  53 (58.9)  37 (41.1) |
| **Comorbidities among patients that reported at least one^1,2^** |  |  |  |
| **Total number of patients, n (%)**  Depression  Generalized anxiety  Diabetes without chronic complications | **97 (100)**  39 (40.2)  36 (37.1)  18 (18.6) | **60 (100)**  21 (35.0)  20 (33.3)  13 (21.7) | **37 (100)**  18 (48.6)  16 (43.2)  5 (<1.0) |

Abbreviations: EU5, France, Germany, Italy, Spain, and United Kingdom; IQR, interquartile range; MOGAD, myelin-oligodendrocyte glycoprotein antibody-associated disease; n, number of PRF responses; PRF, patient record form; SD, standard deviation.

^1^Categories are not mutually exclusive.

^2^Comorbidities with a prevalence in the total sample <2% not reported.

**Table S2.** **Patient demographics and clinical characteristics self-reported by patients with MOGAD, reported overall and by geography**

|  | **Overall** | **EU5** | **US** |
| --- | --- | --- | --- |
| **Age in years at survey completion**  **Total number of respondents, n**  Mean (SD)  Median (Q1, Q3)  Min; max | **66**  33.6 (10.8)  34.0 (22.8; 41.0)  18.0; 65.0 | **56**  33.0 (11.1)  32.0 (22.0; 41.0)  18.0; 65.0 | **7**  38.4 (4.0)  39.0 (35.0; 41.0)  33.0; 45.0 |
| **Sex**  **Total number of respondents, n (%)**  Male  Female | **66 (100)**  27 (40.9)  39 (59.1) | **56 (100)**  25 (42.4)  34 (57.6) | **7 (100)**  2 (28.6)  5 (71.4) |
| **Ethnicity, n (%)^1^**  **Total number of respondents, n (%)** White/Caucasian  African American  Hispanic/Latino  Other | **66 (100)**  63 (95.5)  3 (4.5)  0 (0.0)  0 (0.0) | **56 (100)**  59 (100.0)  0 (0.0)  0 (0.0)  0 (0.0) | **7 (100)**  4 (57.1)  3 (42.9)  0 (0.0)  0 (0.0) |
| **Comorbidities**  **Total number of respondents, n (%)**  None reported  At least one reported  **Comorbidities among patients that reported at least one^1,2^**  **Total number of respondents, n (%)**  Depression  Generalised anxiety  Diabetes without chronic complications | **66 (100)**  48 (72.7)  18 (27.3)  **18 (100)**  8 (44.4)  7 (38.9)  3 (16.7) | **56 (100)**  45 (76.3)  14 (23.7)  **14 (100)**  7 (50.0)  5 (35.7)  3 (21.4) | **7 (100)**  3 (42.9)  4 (57.1)  **4 (100)**  1 (25.0)  2 (50.0)  0 (0.0) |
| **Clinical course, n (%)**  **Total number of respondents, n (%)**  Monophasic  Relapsing | **66 (100)**  36 (54.5)  30 (45.5) | **56 (100)**  31 (52.5)  28 (47.5) | **7 (100)**  5 (71.4)  2 (28.6) |

Abbreviations: EU5, France, Germany, Italy, Spain, and United Kingdom; Q1, Q3: interquartile range; MOGAD, myelin-oligodendrocyte glycoprotein antibody-associated disease; n, number of PSC responses; PSC, patient self-completion form; SD, standard deviation.

^1^Categories are not mutually exclusive.

^2^ Comorbidities with a prevalence in the total sample <2% not reported.

**Table S3. Number and type of tests, scans and assessments performed among patients diagnosed with MOGAD informed by participating neurologists, overall and by geography**

|  | **Overall** | **EU5** | **US** |
| --- | --- | --- | --- |
| **No. tests/scans/assessments conducted to aid MOGAD diagnosis**  **Total number of patients with available information, n**  Mean (SD)  Median (Q1, Q3)  Min; max | **268**  13.7 (7.0)  12.0 (9.0; 19.0)  1.0; 36.0 | **213**  14.8 (7.0)  14.0 (9.0; 20.0)  2.0; 36.0 | **55**  9.6 (5.4)  9.0 (5.0; 12.0)  1.0; 22.0 |
| **Tests/scans/assessments conducted to aid MOGAD diagnosis^1,2^**  **Total number of patients, n (%)**  MOG antibody detection (cell-based assay)  Brain MRI  Spinal cord MRI  Protein electrophoresis (oligoclonal bands)  Physical examination  Complete blood count  Visual evoked potential  Visual field test  Visual acuity test  CSF white cell count  Blood white blood count  CSF viral PCR  Bacterial culture  Blood viral PCR  SPEP  LP opening pressure test  Retinal imaging (fundus photography)  PCR  CSF other PCRb  Lactate  Blood culture  CSF ELISA  Blood ELISA  Blood other PCRb  Head CT scan | **268 (100.0)**  268 (100.0)  242 (90.3)  234 (87.3)  219 (81.7)  193 (72.0)  168 (62.7)  162 (60.4)  155 (57.8)  150 (56.0)  145 (54.1)  133 (49.6)  109 (40.7)  89 (33.2)  85 (31.7)  80 (29.9)  76 (28.4)  74 (27.6)  68 (25.4)  63 (23.5)  59 (22.0)  57 (21.3)  56 (20.9)  54 (20.1)  46 (17.2)  45 (16.8) | **213 (100)**  213 (100)  195 (91.5)  190 (89.2)  182 (85.4)  160 (75.1)  141 (66.2)  141 (66.2)  132 (62.0)  121 (56.8)  119 (55.9)  118 (55.4)  96 (45.1)  78 (36.6)  71 (33.3)  69 (32.4)  54 (25.4)  65 (30.5)  62 (29.1)  56 (26.3)  53 (24.9)  53 (24.9)  50 (23.5)  51 (23.9)  42 (19.7)  43 (20.2) | **55 (100)**  55 (100)  47 (85.5)  44 (80.0)  37 (67.3)  33 (60.0)  27 (49.1)  21 (38.2)  23 (41.8)  29 (52.7)  26 (47.3)  15 (27.3)  13 (23.6)  11 (20.0)  14 (25.5)  11 (20.0)  22 (40.0)  9 (16.4)  6 (10.9)  7 (12.7)  6 (10.9)  4 (7.3)  6 (10.9)  3 (5.5)  4 (7.3)  2 (3.6) |

Abbreviations: ADEM, acute disseminated encephalomyelitis; AQP-4, aquaporin-4; CSF, cerebrospinal fluid; CT, computed tomography; ELISA, enzyme-linked immunosorbent assay; EU5, France, Germany, Italy, Spain, and United Kingdom; Gd, gadolinium; IgG, immunoglobulin G; LP, lumbar puncture; MOGAD, myelin-oligodendrocyte glycoprotein antibody-associated disease; MRI, magnetic resonance imaging; n, number of PRF responses; PCR, polymerase chain reaction; PRF, patient record form; SPEP, serum protein electrophoresis.

^1^Categories are not mutually exclusive.

^2^Tests with a prevalence in the total sample <10% not reported.

Figure S1. Severity of symptoms reported during the initial symptomatic episode among patients with MOGAD, informed by participating neurologists (n=268)

Abbreviations: n, number of PRF responses; PRF, patient record form

No severity data were reported for Babinski sign (n=71) and were therefore excluded from the figure. Symptoms with a prevalence <10% are not presented.
